# Cardiac arrhythmias associated with S1PRMs: a pharmacovigilance study based on real-world data

**DOI:** 10.3389/fphar.2026.1882323

**Published:** 2026-08-18

**Authors:** Siqi Wang, Linli Xie, Pan Ma

**Affiliations:** Department of Pharmacy, The First Affiliated Hospital of Army Medical University (Third Military Medical University), Chongqing, China

**Keywords:** cardiac arrhythmias, disproportionality analysis, drug safety, FAERS, pharmacovigilance, S1PRMs, signal detection

## Abstract

**Background:**

Sphingosine-1-phosphate receptor modulators (S1PRMs) are an effective class of disease-modifying therapies (DMTs) for the treatment of multiple sclerosis (MS). While cardiac arrhythmias such as bradycardia and atrioventricular (AV) block are recognized class effects of S1PRMs, comparative real-world safety profiles across all four approved agents-particularly newer agents with limited post-marketing data-remain insufficiently characterized.

**Methods:**

We conducted a retrospective pharmacovigilance analysis using data from the FDA Adverse Event Reporting System (FAERS) from the fourth quarter of 2010 to the first quarter of 2024. Disproportionality analysis was performed using the Reported Odds Ratio (ROR), Proportional Reporting Ratio (PRR), Bayesian Confidence Propagation Neural Network (BCPNN) and Multi-item Gamma Poisson Shrinker (MGPS). Adverse events were classified using Standardised MedDRA Queries narrow-scope terms and Preferred Terms (PTs). Univariate logistic regression was used to explore the association between demographic factors and arrhythmia reporting.

**Results:**

We identified 2,059 arrhythmia-related adverse events associated with S1PRMs, including 1,860 cases for fingolimod, 153 for siponimod, 38 for ozanimod, and 8 for ponesimod. Patients aged 18–65 and female patients constituted the majority of reports. Disproportionality analysis based signal detection identified heart rate decreased, cardiac flutter, and extrasystoles as prominent signals across S1PRMs.

**Conclusion:**

This pharmacovigilance study provides a comparative real-world overview of arrhythmia-related adverse event signals across four approved S1PRMs. The findings are concordant with the cardiac effects anticipated from the known pharmacology of S1PRMs and reinforce the importance of cardiac monitoring during treatment initiation. As a disproportionality analysis of spontaneous reports, these results reflect statistical associations rather than causal relationships and should be regarded as hypothesis-generating; cross-drug comparisons are further influenced by differential reporting, market duration and cumulative exposure. These results may complement clinical trial data for informed risk-benefit assessment, particularly for newer S1PRMs with limited post-marketing surveillance data.

## 1 Introduction

Multiple sclerosis (MS) is an immune-mediated inflammatory demyelinating disease of central nervous system (CNS), characterized by dissemination in time and dissemination in space (Marcus, 2022; Oh et al., 2018; Koch-Henriksen and Magyari, 2021). The etiology of MS is unknown, but Epstein-Barr virus infection, low serum vitamin D levels, insufficient sun exposure, smoking, and adolescent obesity may be risk factors for MS. Multiple sclerosis is prevalent in patients between the ages of 20 and 40 years, and is more common in women (Yamout et al., 2024). According to the latest MS Atlas, a joint project of the International Multiple Sclerosis Federation and WHO, 2.8 million people worldwide suffered from MS. Since 2013, there has been an increase of 500,000 new cases of MS global. The prevalence and incidence of MS has been steadily increasing worldwide, including in the Middle East and North Africa (MENA) region, over the past few decades (Yamout et al., 2024). The prevalence of MS varies according to geographic regions, with the highest incidence rates (111–300 cases per 100,000 population) reported in the WHO European Region and the Americas (Coetzee and Thompson, 2020).

There is no definitive cure for MS, but a number of disease-modifying therapies (DMTs) that primarily target the inflammatory milieu have been designed to reduce the rate of relapses and the accumulation of disability in patients with MS (Gholamzad et al., 2019). Sphingosine-1-phosphate receptor modulators (S1PRMs), a class of disease-modifying therapies (DMTs), are structurally similar to Sphingosine 1-phosphate (S1P). S1P is a biologically active soluble hemolytic phospholipid signaling molecule. Under normal homeostatic conditions, erythrocytes and endothelial cells are thought to be the major sources of S1P in plasma, whereas mast cells and platelets lead to a localized increase in S1P under noninflammatory and prethrombotic conditions (McGinley and Cohen, 2021; Coyle et al., 2024; Chun et al., 2021). Sphingosine-1-phosphate receptors (S1PRs) are expressed throughout the body and mediate a wide range of biological functions. However, due to the widespread expression of S1PRs on cardiomyocytes and vascular endothelial cells, all drugs related to them will have some cardiovascular effects (Gholamzad et al., 2019; Chun et al., 2021; Roy et al., 2021). Fingolimod, the first approved S1PRMs, non-selectively targets S1PR1, S1PR3, S1PR4 and S1PR5. Some arrhythmic adverse events (AEs) of fingolimod, such as atrioventricular block and sinus bradycardia, have been reported. In early clinical trials of fingolimod, it was found that patients experienced a transient decrease in mean measured heart rate 4–5 h after the first dose of the drug (Chitnis et al., 2018; Mori, 2015). Since studies have shown that this cardiovascular effect may be mediated by transient agonism of S1PR1 or S1PR3, second-generation S1PRMs with low affinity for S1PR3 have been widely developed. However, similar cardiovascular AEs were observed in MS patients receiving siponimod, ozanimod, and other second-generation drugs that reduce S1PR3 binding (Grigorova et al., 2010). Currently, for proved S1PRMs, 6 h of continuous cardiac monitoring after the first dose is recommended to somewhat minimize these side effects (DiMarco et al., 2014).

While the acute cardiac effects of S1PRMs are well-documented from clinical trials, several gaps remain in the current understanding of their real-world arrhythmia risk profiles. First, head-to-head comparative safety data across all four approved S1PRMs (fingolimod, siponimod, ozanimod, ponesimod) from real-world settings are sparse, as most evidence derives from controlled clinical trial environments with selected patient populations. Second, newer agents—ozanimod (approved 2020) and ponesimod (approved 2021)—have limited post-marketing surveillance data, and their arrhythmia signal profiles in routine clinical practice have not been systematically characterized. The FDA Adverse Event Reporting System (FAERS), established in 2012, is one of the largest pharmacovigilance databases in the world, harboring a large number of drug-related AEs for post-marketing safety surveillance. Therefore, this study aimed to conduct a systematic disproportionality analysis of S1PRM-associated arrhythmia signals using the FAERS database to provide comparative real-world safety profiles across all four approved agents, with particular attention to newer S1PRMs with limited post-marketing data.

## 2 Methods

### 2.1 Data sources and procedures

This study is a retrospective, observational pharmacovigilance study using data from the FAERS database. We adhered to the REporting of studies Conducted using Observational Routinely-collected health Data for Pharmacovigilance (READUS-PV) guidelines. The reports of S1PRMs-related adverse reactions submitted from the forth quarter of 2010 when the first approved S1PRM listed to the first quarter of 2024 were extracted from the FAERS database. FAERS was queried by using the generic names “fingolimod” “siponimod” ozanimod” and “ponesimod” with primary suspect drug role. Deduplication was performed using the FDA-recommended case deduplication algorithm: for reports sharing the same CASE number, only the report with the latest receipt date was retained. Additionally, reports with identical combinations of age, sex, event date, and reporter country were flagged and manually reviewed to minimize residual duplicates. For indication-based filtering, all reports were screened for the documented treatment indication (Supplementary Table S1). Among the four S1PRMs studied, only ozanimod carries an additional non-MS approved indication (ulcerative colitis, UC), and 11 of the 38 ozanimod reports (28.9%) explicitly specified UC as the indication; the remaining three drugs are approved for MS only. Because the treatment indication was missing or unrecorded for a substantial proportion of reports across all four drugs, indication-based exclusion could not be applied consistently without disproportionately discarding valid adverse event data, so the complete, indication-unstratified case series was retained for the primary analysis presented in the main text. To directly address this concern for ozanimod, we additionally performed a supplementary descriptive sensitivity analysis restricted to the 27 ozanimod reports without a documented UC indication, presented in Supplementary Table S1; the corresponding residual uncertainty is acknowledged in the Limitations. In total, 2,446 reports were flagged by these criteria, of which 398 (16.27%) were confirmed as duplicates and removed after independent review by two researchers; agreement between the two reviewers was almost perfect (Cohen’s κ = 0.99), and remaining discrepancies were resolved by discussion with a third senior researcher. Two researchers independently used SMQ narrow-scope terms (MedDRA version 26.1) and Preferred Terms (PTs) to classify S1PRMs-related cardiac arrhythmias. Discrepancies were resolved through discussion with a third senior researcher. Patient and drug information were extracted from reports.

### 2.2 Data analysis

Statistical analysis is based on the proportional imbalance method, we used the Reporting Odds Ratio (ROR), Proportional Reporting Ratio (PRR), Bayesian Confidence Propagation Neural Network (BCPNN), and Multi Item Gamma Poisson Shrinker (MGPS) algorithm to study the association between drugs and a specific AE. A signal was considered positive when all four methods met their respective thresholds simultaneously. The equations and criteria for these four algorithms are given in Table 1. These thresholds are the conventional decision criteria recommended for spontaneous-reporting disproportionality analysis and are widely applied in FAERS-based pharmacovigilance studies (ROR: lower bound of the 95% CI > 1 with at least 3 cases; PRR ≥ 2 with χ^2^ ≥ 4 and at least 3 cases; BCPNN: IC025 > 0; MGPS: EBGM05 > 2). Because the four algorithms differ in their sensitivity and in how they shrink estimates for sparse cells, requiring all four criteria to be met simultaneously is a deliberately conservative rule that markedly reduces the probability of chance-driven (false-positive) signals arising from the large number of drug–event pairs examined; it therefore also serves as a built-in safeguard against the multiple-comparisons problem inherent in scanning many SMQs and PTs. Formal Bonferroni or false-discovery-rate correction was not additionally applied because disproportionality analysis is hypothesis-generating rather than confirmatory, and the concordance requirement already imposes a stringent multiplicity control; this is acknowledged in the Limitations.

**TABLE 1:** ROR, PRR, BCPNN, and EBGM methods, formulas, and criterias

Algorithms	Calculation formula	Criteria
ROR	$ROR=\frac{a/c}{b/d}=\frac{ad}{bc}$ $95\%CI=e^{\ln\left(ROR\right)\pm 1.96\sqrt{\frac{1}{a}+\frac{1}{b}+\frac{1}{c}+\frac{1}{d}}}$	(1) a ≥ 3 (2) 95%CI > 1
PRR	$PRR=\frac{a/\left(a+b\right)}{c/\left(c+d\right)}=\frac{a\left(c+d\right)}{c\left(a+b\right)}$ $\chi^{2}=\frac{{\left(\left|ad-bc\right|-\frac{n}{2}\right)}^{2}n}{\left(a+b\right)\left(a+c\right)\left(c+d\right)\left(b+d\right)}$ n = a + b + c + d	(1) a≥3 (2) PRR ≥ 2 (3) $\chi^{2}\geq 4$
BCPNN	$E\left(IC\right)=\log_{2}\frac{\left(C_{xy}+\gamma_{11}\right)\left(C+\alpha \right)\left(C+\beta \right)}{\left(C+\gamma \right)\left(C_{x}+\alpha_{1}\right)\left(C_{y}+\beta_{1}\right)}$	(1) a ≥ 3 (2) IC-2SD > 0
	$V\left(IC\right)=\frac{1}{{\left(\ln2\right)}^{2}}\left\{\left(\frac{C-C_{xy}+\gamma -\gamma_{11}}{\left(C_{xy}+\gamma_{11}\right)\left(1+C+\gamma \right)}\right)+\left(\frac{C-C_{x}+\alpha -\alpha_{1}}{\left(C_{x}+\alpha_{1}\right)\left(1+C+\alpha \right)}\right)+\left(\frac{C-C_{y}+\beta -\beta_{1}}{\left(C_{y}+\beta_{1}\right)\left(1+C+\beta \right)}\right)\right\}$ $\gamma =\gamma_{11}\frac{\left(C+\alpha \right)\left(C+\beta \right)}{\left(C_{x}+\alpha_{1}\right)\left(C_{y}+\beta_{1}\right)}$ $IC-2SD=E\left(IC\right)-2\sqrt{V\left(IC\right)}$ $\alpha_{1}=\beta_{1}=1;\alpha =\beta =2;\gamma_{11}=1;$ $C=a+b+c+d;C_{x}=a+b;C_{y}=a+c;C_{xy}=a$	
EBGM	EBGM = $\frac{a\left(a+b+c+d\right)}{\left(a+b\right)\left(a+c\right)}$ $95\%\text{CI}=e^{\ln\left(\text{EBGM}\right)\pm 1.96\sqrt{\left(\frac{1}{a}+\frac{1}{b}+\frac{1}{c}+\frac{1}{d}\right)}}$	EBGM05 > 2

### 2.3 Statistical analysis

All data were analysed using IBM® SPSS® statistical software. p-value less than 0.05 was considered statistically significant. Age and sex were the only patient-level covariates that were reliably and consistently recorded in the spontaneous reports; accordingly, for each drug we fitted two independent univariate logistic-regression models to assess the respective associations of age and of sex with arrhythmia reporting, rather than a single joint multivariable model. Key clinical confounders that would be required for a fully adjusted multivariable model—such as concomitant rate-limiting medications (beta-blockers, calcium-channel blockers, antiarrhythmics), baseline cardiovascular disease, MS disease duration and activity, and the drug-specific dose-titration schedule (e.g., the 5-day siponimod escalation)—are not captured in a structured, analysable form in FAERS and could therefore not be incorporated; this constraint is discussed explicitly in the Limitations.

## 3 Results and discussion

### 3.1 Descriptive analysis

**FIGURE 1:**
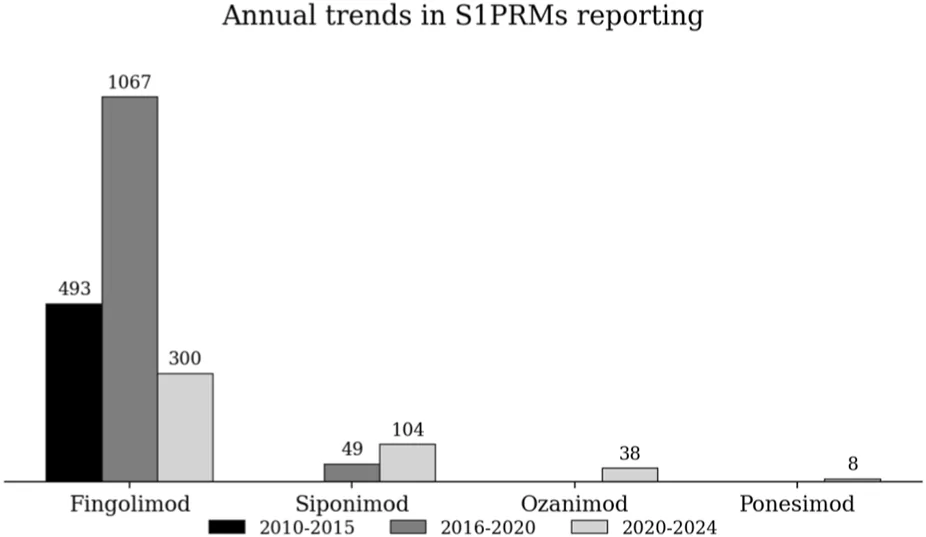
Annual trends in S1PRMs reporting.

We extracted all reported cases from the FAERS database, after deduplication and screening totaling 2,059 cases of S1PRMs-related cardiac arrhythmias were reported, 1,860 of Fingolimod, 153 of Siponimod, 38 of Ozanimod, 8 of Ponesimod. In this study, the reported data were analysed, and the demographic characteristics of the AEs associated are shown in Table 2. Among the adverse events reported, the proportion of women was slightly higher than that of men. In terms of age distribution, although the proportion of patients with unknown age reached 30.69%, the highest proportion of AEs was in the age group of 18–65 years (64.50%). The median age of the four drugs was 45, 56, 43, 60. The country with the most reports is the United States (45.12%), followed by the United Kingdom (20.12%), Germany (6.31%), Canada (2.78%), etc. The temporal distribution of reports for each S1PRM, shown in Figure 1, closely tracks each drug’s market entry: reports for fingolimod (approved 2010) rose sharply and peaked in 2016–2020 before declining, whereas siponimod, ozanimod and ponesimod entered the market considerably later and their cumulative reports remain concentrated in the most recent period; this pattern indicates that the large disparity in raw report counts across drugs primarily reflects differences in marketing duration and cumulative exposure rather than differences in cardiac risk. A total of 282 serious adverse events were mainly reported by fingolimod, including 5 deaths, 257 hospitalization-initial or prolonged, and 18 life-threatening.

**TABLE 2:** Clinical characteristics of reports with S1PRMs-related cardiac arrhythmias from the FAERS database.

Characteristics	Reports			
	Fingolimod	Siponimod	Ozanimod	Ponesimod
N	1,860	153	38	8
Age				
<18	44	-	-	-
18–65	1,197	101	27	3
>65	35	19	1	-
Missing	584	33	10	5
Gender				
Female	1,302	103	25	3
Male	440	44	8	2
Missing	118	6	5	3
Country of the reporter				
United States	655	121	38	6
United Kingdom	30	14	-	-
Germany	96	9	-	1
Canada	62	12	-	0
Reported years				
2010–2015	493	-	-	-
2016–2020	1,067	49	-	-
2020–2024	300	104	38	8
Outcomes				
Death	5	1	-	-
Hospitalization-initial or prolonged	257	7	-	-
Life-threatening	18	1	-	-
Disability	2	-	-	-
Other	​	​	-	-

A dash (–) denotes that no case was reported in that category (i.e., a count of zero), not missing or unavailable data; reports for which age or sex was not provided are tabulated separately in the “Missing” rows. Percentages within each demographic variable are calculated using the column total (N) for each drug as the denominator. For example, for fingolimod the 18–65 age group accounts for 1,197/1,860 (64.4%) of reports and female sex for 1,302/1,860 (70.0%); the corresponding age-group proportions are 101/153 (66.0%) for siponimod, 27/38 (71.1%) for ozanimod and 3/8 (37.5%) for ponesimod.

### 3.2 Standardized signal detection

Table 3 shows the signal intensity of S1PRMs-associated AEs classified by SMQ. Based on our statistical analysis, a total of 22 Standardised MedDRA Queries (SMQ) were affected by adverse events related to fingolimod, 14 by Siponimod, 10 by Ozanimod and 8 by Ponesimod, respectively. Two of the SMQs involved in all four drugs are haematopoietic leukopenia and tachyarrhythmia, term nonspecific. In this study, adverse reactions related to arrhythmias are highlighted (Table 4). Based on our statistical analysis, fingolimod was associated with the highest number and intensity of arrhythmia signals, which may be related to its poor selectivity for S1PR receptors. Fingolimod non-selectively targets S1PR1, S1PR3, S1PR4 and S1PR5, though this observation may also be influenced by its longer market duration and greater cumulative exposure.

**TABLE 3:** ROR of S1PRMs-associated SMQ.

SMQ	Fingolimod	Siponimod	Ozanimod	Ponesimod
Skin premalignant disorders	3.70 (3.58)	2.98 (2.57)	-	-
Optic nerve disorders	3.27 (3.17)	-	-	-
Gastrointestinal premalignant disorders	-	-	2.62 (2.43)	-
Gastrointestinal ulceration	-	-	2.01 (1.91)	-
Haematopoietic leukopenia	2.56 (2.53)	2.39 (2.38)	1.46 (1.30)	1.00 (0.21)
Skin tumours of unspecified malignancy	2.28 (1.81)	2.27 (2.04)	-	-
Conditions associated with central nervous system haemorrhages and cerebrovascular accidents	2.48 (2.37)	1.93 (1.55)	-	-
Optic nerve disorders	-	2.17 (1.79)	1.44 (0.88)	-
Reproductive premalignant disorders	2.38 (1.81)	-	-	-
Disorders of sinus node function	2.39 (2.23)	-	-	-
Retinal disorders	2.20 (2.13)	1.72 (1.49)	-	-
Immune-mediated/autoimmune disorders	1.89 (1.86)	1.67 (1.58)	2.01 (1.91)	-
Breast tumours of unspecified malignancy	1.68 (1.03)	-	-	-
Skin malignant tumours	1.94 (1.85)	-	-	1.39 (0.27)
Liver related investigations signs and symptoms	1.73 (1.68)	-	-	-
Vestibular disorders	1.75 (1.65)	1.45 (1.13)	-	-
Conduction defects	1.75 (1.66)	1.09 (0.77)	-	-
Tachyarrhythmia terms nonspecific	1.43 (1.21)	1.45 (0.86)	1.69 (1.09)	1.28 (0.16)
Hypertension	-	1.59 (1.46)	1.04 (0.85)	-
Noninfectious meningitis	1.43 (1.21)	-	-	-
Ocular motility disorders	1.43 (1.26)	-	-	-
Uterine and fallopian tube malignant tumours	1.23 (1.02)	-	-	-
Depression (excl suicide and self injury)	1.17 (1.12)	-	-	-
Noninfectious encephalitis	1.02 (0.69)	-	-	-
Liver neoplasms benign (incl cysts and polyps)	0.98 (0.53)	1.11 (0.94)	-	-
Gastrointestinal nonspecific inflammation	-	-	0.96 (0.57)	1.57 (0.79)
COVID-19	-	2.35 (2.21)	1.61 (1.42)	1.57 (0.79)
Interstitial lung disease	-	-	-	1.03 (0.10)

**TABLE 4:** Comparison of the constituent ratio of caused arrhythmias by S1PRMs.

S1PRM	Case number	ROR (95% CI)	PRR (χ^2^)	IC (IC_025_)	EBGM (EBGM_05_)
Fingolimod	350	4.11 (3.70, 4.57)	4.05 (800.69)	2.00 (1.87)	4.03 (3.93)
Siponimod	16	3.18 (1.95, 5.20)	3.18 (21.72)	1.45 (0.86)	3.17 (2.68)
Ozanimod	15	3.97 (2.39, 6.60)	3.96 (3.27)	1.69 (1.09)	3.96 (3.45)
Ponesimod	5	6.40 (2.64, 15.53)	6.29 (17.32)	1.62 (0.59)	6.29 (5.40)

### 3.3 PT level signal detection

**FIGURE 2:**
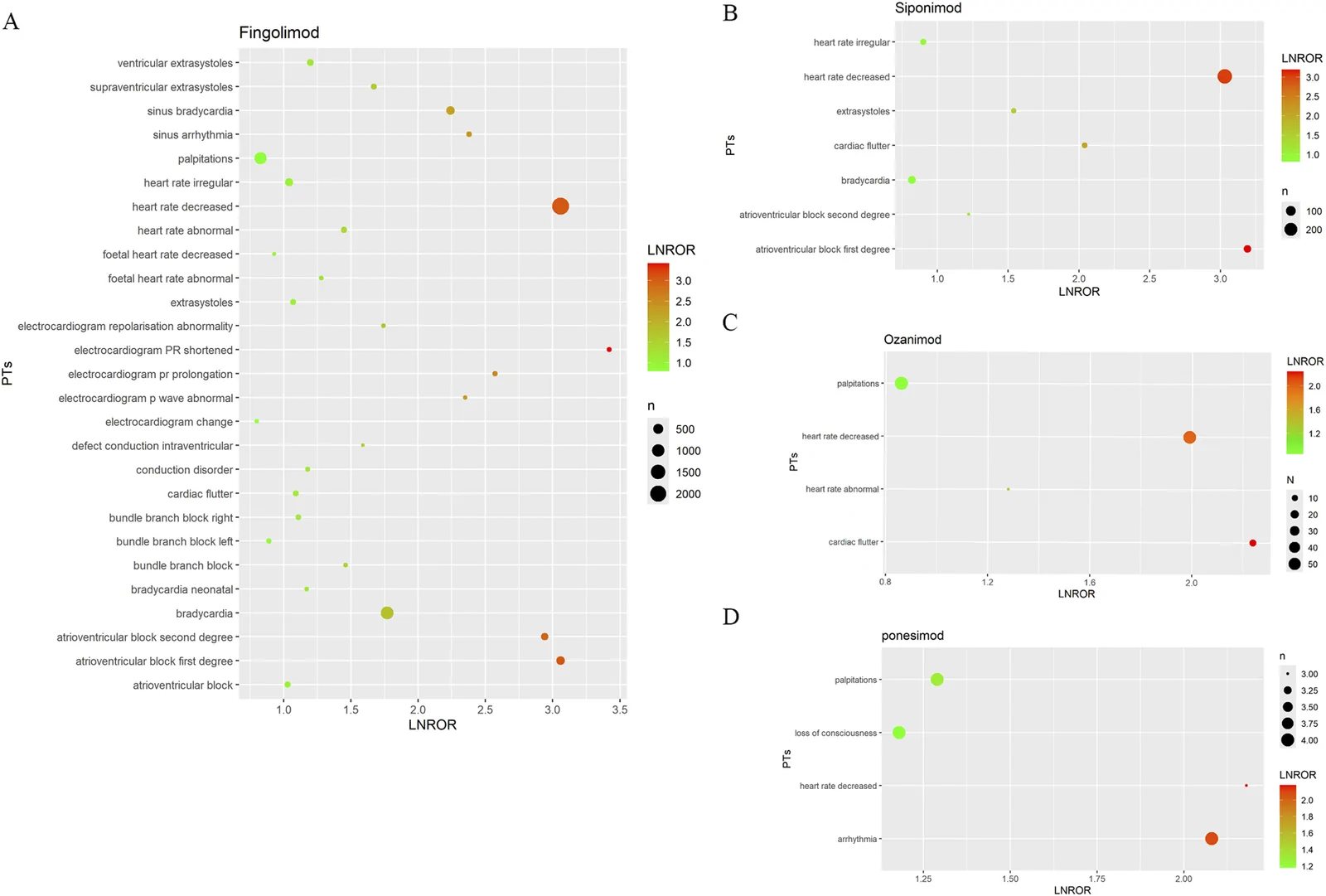
PT level analysis of S1PRMs-associated arrhythmia (**(A)** Fingolimod **(B)** Siponimod **(C)** Ozanimod **(D)** Ponesimod).

**FIGURE 3:**
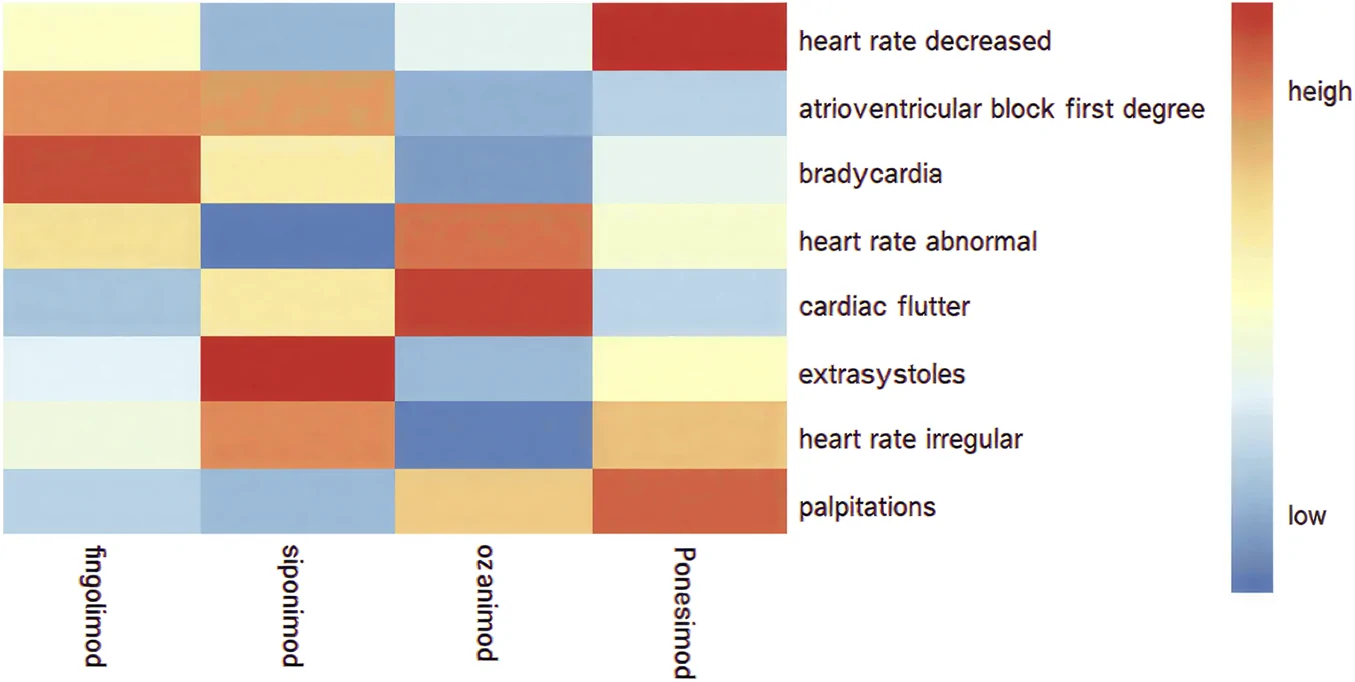
Heatmap of S1PRMs-associated arrhythmia.

Figure 2 shows the PTs for each S1PRM. Fingolimod involved 30 PTs, with the highest signal values primarily including electrocardiogram PR shortened (ROR = 30.54, 95% CI: 20.45–45.62), heart rate decreased (ROR = 21.39, 95% CI: 20.47–22.34), atrioventricular block first degree (ROR = 21.30, 95% CI: 18.97–23.93), atrioventricular block second degree (ROR = 18.85, 95% CI: 16.16–21.98), electrocardiogram PR prolongation (ROR = 13.04, 95% CI: 9.06–18.79) and sinus arrhythmia (ROR = 10.76, 95% CI: 8.18–14.17). Siponimod was associated with 7 PTs, including atrioventricular block first degree (ROR = 24.23, 95% CI: 18.04–32.55), heart rate decreased (ROR = 20.77, 95% CI: 18.41–20.77), cardiac flutter (ROR = 7.65, 95% CI: 4.87–12.02) and extrasystoles (ROR = 4.68, 95% CI: 2.72–8.07). Ozanimod demonstrated 4 PTs, involving cardiac flutter (ROR = 9.38, 95% CI: 5.44–16.17), heart rate decreased (ROR = 7.35, 95% CI: 5.65–9.57), heart rate abnormal (ROR = 3.59, 95% CI: 1.35–9.58) and palpitations (ROR = 2.36, 95% CI: 1.82–3.05). Ponesimod was associated with 4 PTs: heart rate decreased (ROR = 8.85, 95% CI: 2.83–27.67), arrhythmia (ROR = 7.99, 95% CI: 2.98–21.48), palpitations (ROR = 3.62, 95% CI: 1.34–9.72) and loss of consciousness (ROR = 3.24, 95% CI: 1.21–8.72). Notably, all four S1PRMs exhibited a significant signal for heart rate decreased (Figure 3), consistent with the results of previous randomized controlled trials (RCTs). Based on these findings, clinical guidelines emphasize stringent electrocardiographic monitoring protocols during treatment initiation, including baseline ECG evaluation (focusing on PR interval and QTc duration), continuous cardiac monitoring ≥6 h after initial dosing, and contraindication in patients with baseline heart rate <55 bpm or a history of atrioventricular block.

### 3.4 Univariate logistic regression analysis

We performed two independent univariate logistic regression models to assess the respective impacts of age and sex on arrhythmia adverse reactions; results from both models are summarized in Table 5. The analysis results showed that gender had no significant effect on the adverse reaction of arrhythmia caused by the four drugs. Except for ponesimod and ozanimod, the use of S1PRMs was more likely to cause arrhythmia adverse reactions with increasing age. This may be related to the fact that elderly individuals often have cardiovascular underlying diseases such as hypertension and coronary heart disease, as well as the situation of multiple drug combinations. Therefore, when administering medications to elderly patients, it is necessary to fully understand the patients’ medical history, and active electrocardiogram monitoring of heart rate is required after drug administration.

**TABLE 5:** Logistic regression analysis.

Characteristics	Fingolimod		Siponimod		Ozanimod		Ponesimod	
	OR (95% CI)	P	OR (95% CI)	P	OR (95% CI)	P	OR (95% CI)	P
Age	1.26 (1.21, 1.31)	<0.001	1.17 (1.11, 1.24)	<0.001	0.98 (0.96, 1.00)	0.267	1.09 (0.99, 1.21)	0.077
Gender	0.50 (0.24, 1.04)	0.064	1.28 (0.36, 4.53)	0.704	1.25 (0.52, 2.98)	0.610	0.91 (0.08, 9.80)	0.939

## 4 Discussion

Since fingolimod was approved by the U.S. Food and Drug Administration in 2010 as the first sphingosine-1-phosphate receptor modulator for the treatment of relapsed multiple sclerosis, the development of such novel therapeutic compounds has become a pharmacological target of great concern in clinical trial (Vehoff et al., 2017; Kappos et al., 2010). However, S1PR is expressed in a variety of body tissues to regulate multiple physiological and pathological cellular responses involving innate and adaptive immunity, cardiovascular and neurological functions. Due to their widespread expression in cardiomyocytes and vascular endothelial cells, drugs associated with S1PRMs can all produce certain cardiovascular effects. To alleviate this adverse reaction, selective second-generation S1PRMs were subsequently developed (Roy et al., 2021). Nevertheless, similar cardiovascular adverse events were also observed in multiple sclerosis patients treated with siponimod, ozanimod and other second-generation drugs that reduce the binding of S1PR3.

The observation that all four agents produced a heart rate decreased signal despite their differing receptor-selectivity profiles is mechanistically consistent with the central role of S1PR1 in the cardiac effect. The acute negative chronotropic response to S1PRMs is mediated predominantly by agonism of S1PR1 expressed on atrial myocytes and on cells of the cardiac conduction system: receptor activation couples through Gαi/o proteins to the G-protein-gated inwardly rectifying potassium (GIRK/I_KACh) channel, causing membrane hyperpolarization of sinoatrial nodal cells, slowing of the spontaneous depolarization rate and consequent bradycardia and slowed atrioventricular conduction. Because S1PR1 is the obligatory pharmacological target through which all four agents exert their lymphocyte-sequestering efficacy, none of them can be made fully devoid of this on-target cardiac effect, and the bradycardic signal therefore persists across the class regardless of S1PR3 affinity. The rationale for developing S1PR3-sparing second-generation agents was that S1PR3 was thought to contribute additionally to the first-dose effect; however, the human data, including the present analysis, indicate that reducing S1PR3 binding attenuates rather than abolishes the cardiac signal, underscoring the dominant contribution of S1PR1. The progressive desensitization and internalization of S1PR1 with continued dosing (functional antagonism) further explains why the effect is largely transient and confined to treatment initiation, providing the pharmacological basis for both the recommended first-dose monitoring and the dose-titration schedules used for the newer agents.

The present study provides a comparative analysis of arrhythmia-related adverse event signals across all four approved S1PRMs using the FAERS database, encompassing over 13 years of post-marketing data. While individual cardiac safety profiles of S1PRMs are well-described in product labeling and clinical trial literature, this study contributes a systematic cross-drug comparison using real-world spontaneous reporting data, with particular emphasis on the newer agents ozanimod and ponesimod, for which post-marketing surveillance data remain limited.

Our findings are broadly consistent with the known pharmacological profiles of S1PRMs. In this study, we identified 2,059 arrhythmia-related adverse events associated with S1PRMs, including 1,860 cases for fingolimod, 153 for siponimod, 38 for ozanimod, and 8 for ponesimod. The proportion of reports from women is higher, and the proportion of patients aged 18–65 is even greater. This may be related to the fact that the prevalence of multiple sclerosis varies by gender, with epidemiological studies finding a total ratio of 3:1 for women to men. The pronounced gradient in case numbers across the four agents—from 1,860 reports for fingolimod to only 8 for ponesimod—is best explained by differences in market availability rather than by intrinsic differences in per-patient arrhythmia risk. Fingolimod has been marketed since 2010 and has by far the largest cumulative patient exposure, whereas ozanimod and ponesimod were approved only in 2020 and 2021, respectively, and have correspondingly short post-marketing windows and limited clinical uptake. Spontaneous report counts scale with cumulative exposure and time on the market, so the low absolute number of ponesimod reports reflects its recent approval and small exposed population, not a demonstrably safer cardiac profile. This disparity also limits the statistical power to detect signals for the newer agents and should be borne in mind when comparing signal counts across drugs. In addition, the study described a high proportion of AEs in the United States (45.12%), United Kingdom (20.12%), Germany (6.31%), which may be related to the fact that these agents are currently marketed predominantly in Western countries. This geographic concentration should be interpreted with caution: the predominance of reports from the United States and United Kingdom is also strongly shaped by differences in national pharmacovigilance infrastructure and reporting culture—most notably the mandatory manufacturer reporting requirements and the high spontaneous-reporting rate in the United States—rather than reflecting a genuinely higher per-patient risk in these regions. Consequently, the geographic distribution of reports limits the generalizability of these signals to populations and prescribing settings outside North America and Western Europe.

Based on our statistical analysis, a total of 22 SMQs were identified for adverse events related to fingolimod, 14 to Siponimod, 10 and 8 to Ozanimod, Ponesimod, respectively. The results showed that fingolimod was associated with the highest signal intensity for arrhythmia, while there was no significant difference in the association strength between selective second-generation S1PRMs and arrhythmia. The PTs with the highest signal value for fingolimod were bradycardia and first- and second-degree AV block. This is consistent with the adverse reactions observed in clinical trials (Zhao et al., 2021).

After the first administration of fingolimod, the heart rate usually shows a brief decrease, with the lowest point typically occurring 4–5 h after administration. The pooled data analysis of the phase III trial of fingolimod showed that only 0.6% of the patients had symptomatic bradycardia. The incidence of first-degree atrioventricular block was 4.7%, while the incidence of second-degree atrioventricular block (Mobitz type I) was lower, at 0.2% (DiMarco et al., 2014). Importantly, the disproportionality signals observed here for fingolimod are qualitatively concordant with these phase III trial data: the same event categories that occurred most frequently in the pooled trials—bradycardia/heart rate decreased and first- and second-degree atrioventricular block—are precisely those that emerged as the strongest FAERS signals. The two data sources are therefore complementary rather than directly comparable. Randomized controlled trials provide true incidence rates within selected populations (e.g., symptomatic bradycardia in 0.6% of patients), whereas spontaneous-reporting disproportionality analysis lacks a denominator and instead characterizes which events are reported more often than expected relative to the rest of the database; the magnitude of an ROR cannot be equated with an event rate. The agreement in the type of cardiac events across the two approaches strengthens confidence that the FAERS signals reflect a genuine class effect rather than reporting artefact, while the absence of incidence estimates from FAERS reinforces the continued value of the controlled-trial data for quantifying absolute risk. A notable early post-marketing case involved a 48-year-old patient who died 5 months after fingolimod initiation, with autopsy findings suggesting ventricular arrhythmia as the cause of death (Lindsey et al., 2012). While this case is historical, it underscores the importance of ongoing cardiac vigilance. Studies have found that bradycardia occurs in 4.4% of users of siponimod. It is usually asymptomatic and resolves spontaneously within 24 h without the need for intervention. The five-day dose escalation method can be adopted for the use of siponimod to alleviate the reducing effect of siponimod on heart rate in the initial stage of use. If the patient has no history of heart disease, there is no need for monitoring in a medical institution during treatment (Chun et al., 2021; Selmaj et al., 2013). Ozanimod and ponesimod demonstrated lower arrhythmia signal intensity in our analysis. No clinically significant cardiac conduction abnormalities emerged as strong signals, though both agents were associated with heart rate decreased. To mitigate the impact on heart rate during the first administration, the same dose-escalation regimen could be used (Cohen et al., 2019; Cohen et al., 2016).

Furthermore, this study explored the association of age and gender with adverse event reporting of arrhythmia. The results of the univariate logistic regression analysis showed that gender did not significantly associate with arrhythmia reporting for the four drugs. Except for ponosimod and ozanimod, the reporting of arrhythmia increased with age when using S1PRMs, possibly due to the presence of cardiovascular conditions such as hypertension and coronary heart disease, as well as the use of multiple medications. This suggests that clinicians should conduct a comprehensive assessment of potential risk factors before prescribing these drugs and closely monitor the patient’s heart rate and blood pressure during treatment.

### 4.1 Limitations

This study has several important limitations inherent to pharmacovigilance analyses using spontaneous reporting databases. First, FAERS data are subject to reporting bias, including notoriety bias (increased reporting of well-known adverse effects) and the Weber effect (declining reporting rates over time as a drug matures on the market). Given that the cardiac effects of fingolimod have been well-publicized since its approval, the disproportionality signals for fingolimod may be inflated compared to newer agents. Second, the absence of denominator data (total number of patients exposed) in FAERS prevents the calculation of true incidence rates, and disproportionality measures reflect statistical associations rather than causal relationships. Third, we were unable to adjust for important confounders such as concomitant medication use (e.g., beta-blockers, calcium channel blockers), baseline cardiovascular disease, or disease severity in the primary disproportionality analysis. The univariate logistic regression provides only limited insight into these confounding factors. Fourth, because treatment indication was incompletely documented in FAERS, the primary analysis could not be stratified by indication; although only ozanimod carries a non-MS approved indication (ulcerative colitis) among the four S1PRMs studied, and a supplementary sensitivity analysis restricted to non-UC ozanimod reports is provided (Supplementary Table S1), residual confounding by indication cannot be fully excluded from the primary, indication-unstratified dataset. Finally, as this is a descriptive pharmacovigilance study, the findings should be interpreted as hypothesis-generating and cannot establish causal relationships between S1PRMs and specific arrhythmia outcomes. Taken together, these constraints mean that our disproportionality signals should be interpreted as hypothesis-generating rather than as a validated, exposure-adjusted ranking of comparative cardiac risk. In particular, the absence of an exposure denominator, the multiplicity inherent in screening many drug–event pairs, the lack of onset-timing data, and the report-level (rather than subtype-specific) capture of serious outcomes each limit how directly these findings can be compared across agents or translated into clinical risk stratification.

## 5 Conclusion

In summary, this study systematically analyzed the arrhythmia-related adverse event signals associated with S1PRMs based on real-world data from FAERS. Through comparative analysis across all four approved S1PRMs, arrhythmia-related adverse reactions were reported predominantly in women and patients around 45 years of age. Fingolimod exhibited the highest signal intensity for arrhythmia events, consistent with its non-selective S1PR binding profile and longer market duration. These results are derived from spontaneous-reporting disproportionality analysis and should be regarded as hypothesis-generating associations rather than measures of causal risk or true incidence, and cross-drug comparisons remain confounded by differential reporting and unequal market exposure. These findings complement existing clinical trial evidence and may inform clinical decision-making, particularly for newer S1PRMs where post-marketing data remain limited. To validate the signals identified here, we propose a prospective, multicentre cohort study (or a nested analysis within existing MS disease registries) that enrols patients initiating each of the four S1PRMs, applies standardized first-dose and longitudinal ECG monitoring, and captures denominator and exposure data together with key confounders (concomitant rate-limiting drugs, baseline cardiovascular disease, MS disease characteristics and the drug-specific titration schedule). Such a design would permit exposure-adjusted, multivariable estimation of comparative cardiac risk and time-to-event characterization of the first-dose window, thereby converting the present hypothesis-generating signals into quantitative risk estimates to guide monitoring protocols in routine clinical practice.

## Data Availability

The original contributions presented in the study are included in the article/Supplementary Material, further inquiries can be directed to the corresponding authors.
