# Supplementary material for: Cardiac arrhythmias associated with S1PRMs: a pharmacovigilance study based on real-world data
Source: Front Pharmacol. 2026 Aug 18;17:1882323. doi: 10.3389/fphar.2026.1882323 (PMC13529548; doi:10.3389/fphar.2026.1882323)
Supplement: Supplementary file 1 [file Table1.docx]

# Supplementary Data

# Table S1 Clinical characteristics of reports with ozanimod-related cardiac arrhythmias from the FAERS database

| Characteristics | Reports |
| --- | --- |
| Reports | Ozanimod |
| N | 27 |
|  |  |
| Age |  |
| ＜18 | - |
| 18-65 | 20 |
| ＞65 | 1 |
| Missing | 6 |
| Gender |  |
| Female | 18 |
| Male | 5 |
| Missing | 4 |
| Country of the reporter |  |
| United States | 27 |
| United Kingdom | - |
| Germany | - |
| Canada | - |
| Reported years |  |
| 2010-2015 | - |
| 2016-2020 | - |
| 2020-2024 | 27 |
| Outcomes |  |
| Death | - |
| Hospitalization-Initial or Prolonged | - |
| Life-Threatening | - |
| Disability | - |
| Other | - |

As shown in Table S1, the documented indications of fingolimod siponimod and ponesimodmodulators were predominantly multiple sclerosis (MS) or unrecorded. In contrast, 11 ozanimod reports (28.9% of all ozanimod entries) specified ulcerative colitis (UC) as the treatment indication. Patients with MS and UC differ substantially in age profiles, cardiovascular comorbidities, and concomitant medications, all of which are established risk modifiers for arrhythmia. We performed descriptive analyses after filtering out clearly non-MS indications as suggested; however, a large proportion of FAERS records lacked documented treatment indications and were retained to avoid excessive loss of adverse event data.
